# Multi‐Cohort Analysis Reveals Genetic Predispositions to Clonal Hematopoiesis as Mutation‐Specific Risk Factors for Stroke

**DOI:** 10.1002/ggn2.202400047

**Published:** 2025-02-08

**Authors:** Shuyang Lin, Yang E. Li, Yan Wang

**Affiliations:** ^1^ Department of Hematology Washington University School of Medicine in St Louis St. Louis MO 63110 USA; ^2^ Department of Genetics Washington University School of Medicine in St Louis St. Louis MO 63110 USA; ^3^ Department of Neurosurgery Washington University School of Medicine in St Louis St. Louis MO 63110 USA; ^4^ Department of Neurology Washington University School of Medicine in St Louis St. Louis MO 63110 USA

**Keywords:** clonal hematopoiesis, hematological traits, mendelian randomization, modified rankin scale, stroke

## Abstract

Recent observational studies have found an association between Clonal Hematopoesis (CH) and strokes but with incomplete results. This study aims to comprehensively characterize mutation‐specific effects of CH on ischemic and hemorrhagic stroke subtypes and 90‐day functional outcomes through publicly available genome‐wide association study (GWAS) cohorts and Mendelian Randomization. TET2 is associated with an increased risk of overall stroke (OR = 1.06, *P* = 0.02), ischemic stroke (OR = 1.05, *P* = 0.03), transient ischemic attack (OR = 1.07, *P* = 0.01) and small vessel stroke (OR = 1.29, *P* = 0.01), as well as adverse 90‐day modified Rankin scale (mRS ≥ 3) before (OR = 1.34, *P* = 0.005) and after adjusted for age, sex, and stroke severity (OR = 1.30, *P* = 0.02). While the presence of any CH mutation is associated with intracerebral hemorrhage (ICH) (OR = 1.21, *P* = 0.02), specific mutations, SRSF2 and ASXL1 are protective against ICH (OR = 0.9, *P* = 0.04) and nontraumatic subarachnoid hemorrhage (OR = 0.92, *P* = 0.03), respectively. In conclusion, the study provided genetic evidence that TET2 is strongly associated with an increased risk of ischemic stroke and poor functional recovery. Future studies clarifying the relationship between CH and hemorrhagic stroke subtypes are needed.

## Graphical abstract

This study comprehensively evaluated the differential effect of clonal hematopoiesis (CH) mutations on the risk of various stroke subtypes and functional recovery. It shows that TET2 is associated with small vessel stroke possibly via a pro‐inflammatory pathway.

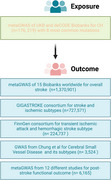

## 1 Introduction

Clonal hematopoiesis (CH) is an age‐related disorder defined by clonal expansion of hematopoietic stem cells.^[^ ^1^, ^2^ ^]^ The prevalence of CH increases with age, affecting over 25% and 62% of individuals over the age of 70 and 80, respectively.^[^ ^3^, ^4^ ^]^ Traditionally considered benign with a low risk of malignant transformation, CH mutations of epigenetic regulators, such as TET2, DNMT3A, and ASXL1, are now linked to increased risks of coronary artery disease and myocardial infarction.^[^ ^5^, ^6^, ^7^ ^]^ These risk factors potentially act through elevated inflammatory profiles causing accelerated atherosclerosis.^[^ ^7^, ^8^, ^9^, ^10^ ^]^ CH presence and clone size are also linked to an increased risk of ischemic and hemorrhagic strokes in observational studies.^[^ ^6^, ^7^ ^]^ However, those results are limited by the types of CH mutations examined, such as a lack of splicing mutations SF3B1 and SRSF2. Additionally, they did not include all stroke subtypes and have a lack of insight into stroke outcomes. Thus, there is a pressing need for further studies with more robust methods on the association of CH mutations with various stroke subtypes and outcomes.

Genome‐Wide Association Studies (GWAS) have been instrumental in identifying genetic risk variants associated with human health and diseases to validate observational findings genetically.^[^ ^11^, ^12^ ^]^ Mendelian Randomization (MR) is an increasingly popular epidemiological tool that provides more robust evidence than observational studies. MR leverages genetic variants identified from GWAS as instrumental variables to infer relationships between risk factors (exposure) and disease (outcome).^[^ ^13^ ^]^ This method is particularly advantageous for dissecting the functional impact of CH, given that it has a strong genetic component. It can also provide helpful insights into polygenic diseases like stroke.

In this MR study, we utilized summary data from large‐scale biobanks and consortiums to investigate the association between CH and stroke.^[^ ^14^ ^]^ First, through GWAS and MR analysis, we comprehensively assessed the effects of common CH mutations on ischemic stroke, hemorrhagic stroke, and post‐ischemic stroke recovery. Second, we offered potential mechanistic pathways underlying specific CH mutation and ischemic stroke through mediation and colocalization analyses. Ultimately, we aim to provide novel insight into the association between CH and both ischemic and hemorrhagic stroke, setting the stage for further investigations of common downstream pathways or mutation‐specific mechanisms.

## 2 Experimental Section

### 2.1 Data Acquisition for Clonal Hematopoiesis

The overall design of the study is shown in Figure S1 (Supporting Information). To acquire the GWAS statistics for CH with different driver mutations (exposure), it acquired GWAS data from the UK Biobank and from the deCODE consortium (total n = 176219).^[^ ^15^, ^16^ ^]^ To extract instrumental variables from those GWAS, it computed the standard error (SE) and effect size (β) by using the basic R package and the formula $SE=\frac{\left|\beta \right|}{Z}\;,\beta =\log\left(\mathrm{OR}\right),Z=qnorm\left(1-\frac{P}{2}\right).$ After obtaining the SE and β, it selected single nucleotide polymorphisms (SNPs) associated with each CH subtype by filtering for $P<5\times 10^{-6}$, accounting for multiple comparisons in GWAS and ensuring the resulting SNPs satisfy the relevance assumption of Mendelian Randomization.^[^ ^17^, ^18^ ^]^ Next, to minimize the effect of linkage disequilibrium, it clumped the SNPs at R2 < 0.001, and kb = 10000 (default parameter) using European ancestry reference.

### 2.2 Data Acquisition for Stroke Subtype and Functional Outcome

An overview of data sources for ischemic and hemorrhagic strokes and their subtypes are shown in **Table** **1**. Data on the combined ischemic stroke and hemorrhagic stroke diagnosis were acquired from meta‐analysis GWAS of the Global Biobank Meta‐Analysis Initiative (GBMI), which included 16 biobanks: BBJ, BioME, BioVU, CCPM, ESTBB, FinnGen, GNH, GS, HUNT, Lifelines, MGB, MGI, QSKIN, TWB, UCLA.^[^ ^19^ ^]^ Additional GWAS and their corresponding stroke phenotypes are listed in Table 1.^[^ ^16^, ^20^, ^21^ ^]^ Stroke diagnosis and subtype classification were made by the original investigators of each GWAS and provided in a summary‐level format in each GWAS database. Briefly, a stroke diagnosis was made by experienced neurologists based on participant self‐reports, medical records, and neuroimaging. Ischemic stroke subtypes were classified based on the Trial of Org 10172 in Acute Stroke Treatment (TOAST) classification (**Table** **2**).^[^ ^22^ ^]^ Hemorrhagic stroke subtypes were classified based on location. Participants without a stroke diagnosis were included as controls. All outcome GWAS were based on European ancestry except for GBMI, which was based on trans‐ancestry.

**Table 1:** Sources of GWAS used in this study.

Data sources for studied phenotypes					
Study	Phenotype	Cases	Controls	PMID	Adjustment
Exposure					
UKB + deCODE	CH	16,306	159,913	37932435	age, sex, ancestry
INTERVAL	Neutrophil + Esosinophil	173,480	N/A	27863252	age, sex, ancestry
Outcome ‐ All stroke					
GBMI	Ischemic and Hemorrhagic Stroke	60,176	1,310,725	36777996	age, sex
Outcome ‐ Ischemic stroke and its subtypes					
FinnGen	TIA	19,929	409,280	36653562	age, sex, ancestry
FinnGen	Ischemic Stroke	24,627	404,582	36653562	age, sex, ancestry
GIGASTROKE	large artery stroke	6,399	1,234,808	36180795	age, sex, ancestry
GIGASTROKE	small vessel stroke	6,811	1,234,808	36180795	age, sex, ancestry
GIGASTROKE	Cardioembolic stroke	10,804	1,234,808	36180795	age, sex, ancestry
Outcome‐Hemorrhagic stroke and its subtypes					
FinnGen	Non Traumatic Subarachnoid Hemorrhage	3,532	425,677	36653562	age, sex, ancestry
FinnGen	Intracerebral hemorrhage	4,056	38,853	36653562	age, sex, ancestry
Chung et al	ToallCH+SVS	6,255	233,058	31430377	age, sex, ancestry
Chung et al	Lobar ICH+SVS	5208	233,058	31430377	age, sex, ancestry
Chung et al	Non‐lobar ICH+SVS	5,468	233,058	31430377	age, sex, ancestry
Outcome ‐ Ischemic stroke functional outcome					
Soderholm et al	mRS≥3	1796	2567	30796134	age, sex, initial stroke severity measure by NIH stroke scale, and ancestry

**Table 2:** Commonly used abbreviations in this study.

Commonly used abbreviations		Diagnostic critera
IS	Ischemic Stroke	TOAST
TIA	Transient Ischemic Attack	TOAST
LAS	Large Artery Stroke	TOAST
SVS	Small Vessel Stroke	TOAST
CMS	Cardioembolic Stroke	TOAST
ICH	Intracerebral hemorrhage	Location
SAH	Subarachnoid hemorrhage	Location
mRS	modified Rankin sore	90 days post stroke

The functional outcomes of ischemic stroke were obtained from Soderholm et al.^[^ ^23^ ^]^ Patients were interviewed by the original investigators 90 days post stroke. The outcomes were measured using the modified Rankin Scale (mRS, Table 2).^[^ ^24^ ^]^ The original GWAS analysis dichotomized the scores into two groups: 1) mRS 0–2: no symptoms to slight disability and 2) mRS 3–6: ranges from moderate disability to death. The mRS were scored 90 days post the incidence of stroke by an experienced neurologist and retrieved from electronic health records. The original GWAS also provided adjusted mRS for age, sex, initial stroke severity measured by NIH stroke scale, and ancestry.

### 2.3 Mendelian Randomization

Mendelian Randomization was performed according to standard practice using the TwoSampleMR package (version 0.5.9). Briefly, after selecting the SNPs as instrumental variables from the exposure according to the criteria above, it extracted the corresponding SNPs from the outcome. Next, it harmonized exposure and outcome by aligning the reference allele to the human genome reference sequence. The harmonization function was a build‐in function of the TwoSampleMR package. It performs covariate harmonization by 1) correcting the effect/reference allele, 2) minimizing strand bias, 3) removing palindromic SNPs, and 4) removing SNPs with incompatible alleles.^[^ ^25^ ^]^ Harmonization helps to satisfy the exclusion restriction assumption, which states that the SNPs affect outcomes through exposure. Next, the random effects two‐sample inverse‐weighted variance (IVW) method, a widely used MR method, was used to estimate the effect of exposure on the outcome. IVW was a two‐tailed Wald test, weighted by inverse variance, that generates P value from the regression coefficient representing the causal estimate. An IVW estimate with a *P* < 0.05 was interpreted as statistically significant.^[^ ^26^, ^27^ ^]^ To capture all possible associations between CH and stroke, it categorized the strength of association generated from MR analyses as weakly, moderately, and strongly, corresponding to *P* ≤ 0.1, ≤ 0.05, and ≤ 0.01, respectively.

### 2.4 Sensitivity Analyses

Given that IVW estimates can be biased by heterogeneity effects, it examined the heterogeneity of SNPs used to perform the initial MR analysis by performing Cochran's Q‐test. Similarly, it performed the MR Egger's intercept test to evaluate the potential pleiotropy effects of the instrumental variables. *P* > 0.05 indicated the absence of heterogeneity and pleiotropy, thereby satisfying the independence assumption.

### 2.5 Mediation Analysis

Neutrophil and eosinophil count GWAS were obtained from the INTERVAL consortium and examined as a potential mediator for CH on ischemic stroke.^[^ ^28^ ^]^ The mediator effect was calculated using the two‐step method. Two‐step mediation analysis requires 1) forward association between exposure and the outcome 2) forward association between mediator and the outcome 3) forward exposure between exposure and mediator 4) no reverse association between outcome and exposure.^[^ ^29^ ^]^ The mediator effect was calculated using the product method. The mediator effect was interpreted as the extent to which the effect of the exposure on the outcome can be explained by the mediator.

### 2.6 Colocalization

MR results can still be susceptible to LD despite a negative pleiotropy test. Colocalization analysis was performed to further reduce biases introduced by LD as previously described.^[^ ^17^ ^]^ Briefly, Coloc.abf function was performed with the Bayesian model using the coloc package (version 5.2.1). Window size for the most significant exposure‐outcome SNP pair was set at ± 500 kb. Default priors, with *P1* as 1 × 10^−4^, *P2* as 1 × 10^−4^, and *P12* as 1 × 10^−5^ were used. Locuszoom package (version 1.4) was used for visualization. A H4 value ≥ 0.8 indicates colocalization of risk variants.

### 2.7 Statistical Analysis

It have outlined the method used for the pre‐processing of data in the preceding sections as well as the respective sample size (see data acquisition and Table 1). It have presented the MR analysis results as odd ratios and P values. The assumptions of MR analysis and the applications of inverse‐variance‐weighted tests have been discussed in the Mendelian Randomization section. Similarly, details for sensitivity analysis and mediation analyses have been discussed in the preceding sections. All data analyses were performed with R (version 4.4.1) with specific package detailed.

## 3 Results

### 3.1 GWAS from UK Biobank and deCODE Consortium Identified Eight CH Mutations as Exposure

We have included a table of commonly used abbreviations of stroke subtypes in Table 2. The MR analysis included CH with different driver mutations from the meta‐analysis of UK Biobank and deCODE consortium as exposure. We identified 8 distinctive mutations (ASXL1, DNMT3A, GNBI, JAK2, PPM1D, SF3B1, SRSF2, TET2) and categorized the presence of any CH mutation as ALL.^[^ ^15^ ^]^ The most common mutations of CH are DNMT3A, TET2, and AXSL1, which are epigenetic regulators, followed by PPM1D involved in DNA damage repair, JAK2 and GNB1 involved in cellular signaling, and the less common SF3B1 and SRSF2 mutations with roles in alternative splicing.^[^ ^30^ ^]^ The exact role of these mutations in the pathogenesis of CH and related physiological impact is not fully elucidated. After selecting SNPs with genomic significance and clumping for LD, 95, 11, 22, 8, 7, 13, 4, 4, and 17 instrumental variables were extracted to perform MR analysis for ALL, ASXL1, DNMT3A, GNB1, JAK2, PPM1D, SF3B1, SRSF2, TET2, respectively. Detailed MR results can be found in supplemental tables.

### 3.2 TET2 is Associated with a Higher Risk of Overall Stroke

**Figure 1:**
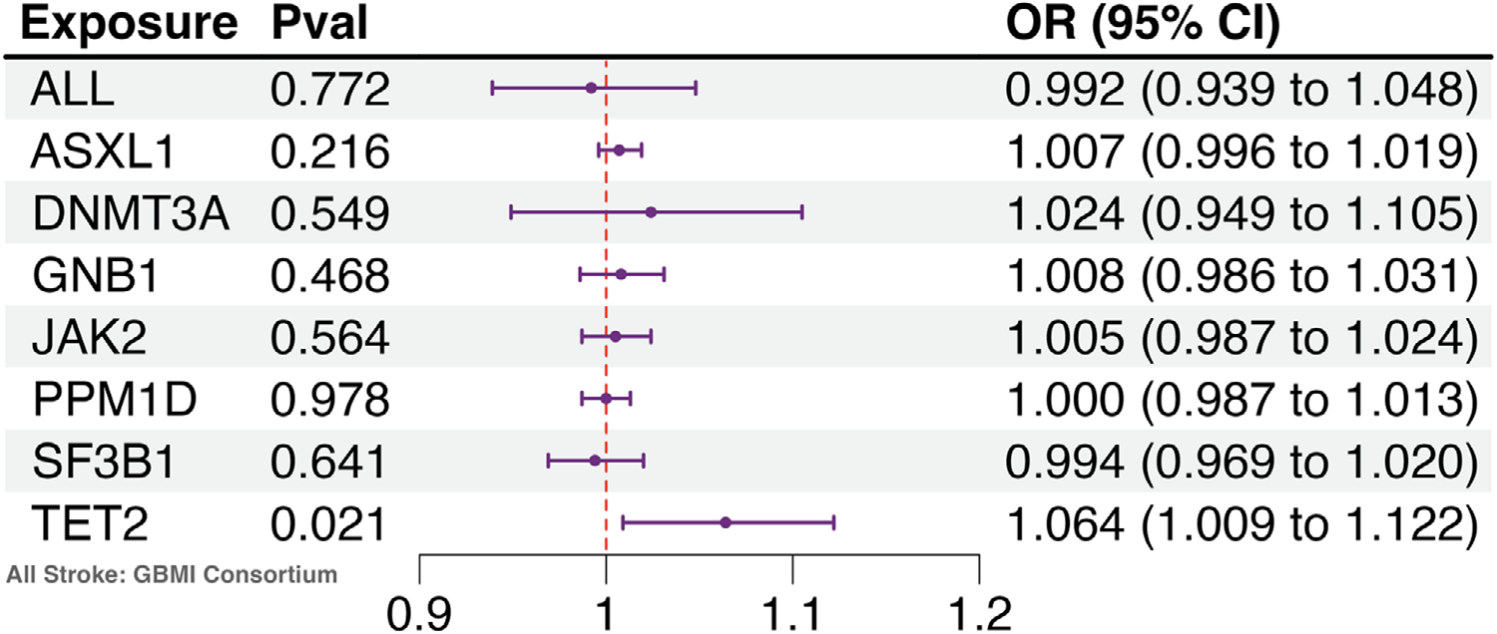
Forest plot showing the MR analysis results between each CH driver mutation and overall stroke risk from GBMI. OR = Odd Ratio, Pval = P value. Forest plot showing the MR analysis results between each CH driver mutation and overall stroke risk from GBMI (N = 1, 370901, P = 0.021 for TET2, assessed by IVW test). Only TET2 CH was found to be significantly associated with overall stroke risk.

We used GBMI GWAS to evaluate the effect of CH on overall stroke, including both ischemic and hemorrhagic strokes. Only TET2 was associated with a moderately increased risk for overall stroke (OR = 1.064, *P* = 0.021). Other mutations were not associated with overall stroke risk (**Figure** **1**; Table S1, Supporting Information). No significant heterogeneity (*P* = 0.14) and pleiotropy effects (*P* = 0.08) were identified (Table S2, Supporting Information). Instrumental variables for SRSF2 were absent in GBMI GWAS and MR analysis was not performed.

### 3.3 TET2 is Associated with Ischemic Stroke and its Subtypes

**Figure 2:**
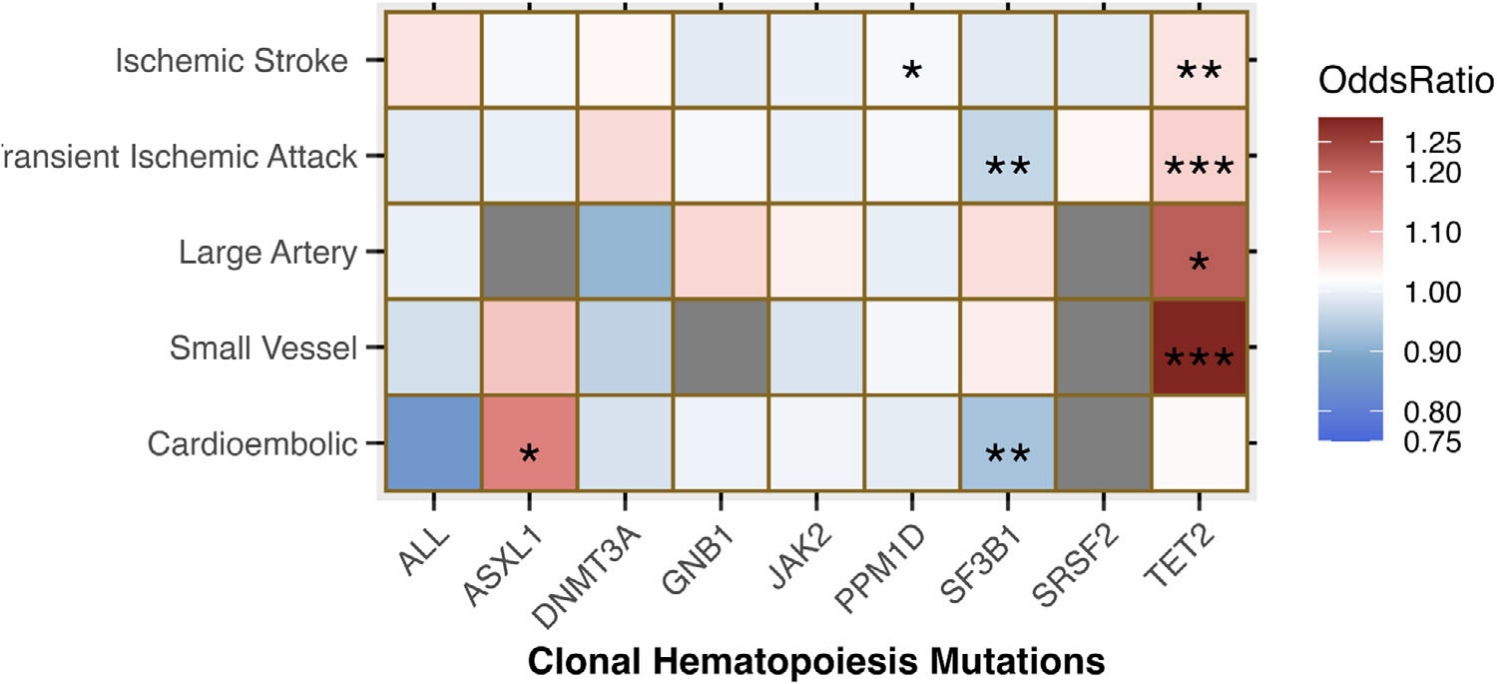
TET2 CH is associated with high risk for ischemic stroke from GIGASTROKE and FinnGen consortia. Heatmap showing the result summaries of 45 independent MR analyses. Gray box indicates insufficient SNPs for the analyses. The color gradient from blue to red indicates OR with blue being < 1, white being 1, and red being > 1. * indicates weak association (*p* ≤ 0.1) ** indicates moderate association (*p* ≤ 0.05), *** indicates strong association (*p* ≤ 0.01). N = 1241207 for GIGASTROKE, N = 429209 for FinnGen. P values are assessed by IVW test.

To further dissect the differential effect of different CH driver genes on ischemic stroke and its subtypes, we used the GIGASTROKE consortium and FinnGen R10 consortium to assess the effect of CH with ischemic stroke and transient ischemic attack (TIA). TET2 was moderately associated with ischemic stroke (OR = 1.05, *P* = 0.03) and strongly associated with TIA (OR = 1.07, *P* = 0.01). By stroke subtypes, TET2 was strongly associated with small vessel stroke (SVS) (OR = 1.29, *P* = 0.01), weakly associated with large artery stroke (LAS) (OR = 1.21, *P* = 0.06), and not with cardioembolic stroke (**Figure** **2**; Tables S3 and S6, Supporting Information). CH mutation SF3B1 was protective against cardioembolic stroke (CMS) (OR = 0.93, *P* = 0.02, Figure 2; Table S3, Supporting Information). No heterogeneity or pleiotropy effects were identified (Tables S4 and S7, Supporting Information). Lastly, we were unable to perform MR for SRSF2 on LAS, SVS, and CMS, ASXL1 on LAS, and GNB1 on SVS as those SNPs were absent from the outcome GWAS (Figure 2).

### 3.4 TET2 Effect on Small Vessel Stroke is Mediated by Granulocyte Cell Count

Prior studies have shown that TET2 mutations in hematopoietic stem cells increase the inflammatory properties of myeloid cells, and inflammation plays an important role in ischemic brain damage.^[^ ^8^, ^10^, ^31^, ^32^, ^33^ ^]^ Given that TET2 loss of function mutation further complicates the development and function of neutrophils, we examined if neutrophil and eosinophil cell count [(Neu + Eos) #] was a mediator of TET2 on stroke using the INTERVAL consortium (Figure S2A, Supporting Information). TET2 was associated with increased (Neu + Eos) # (OR = 1.23, *P* <0.01) and (Neu + Eos) # was associated with SVS (OR = 1.26, *P* = 0.03). TET2 remained independently associated with SVS after controlling for (Neu + Eos) # (OR = 1.29, *P* = 0.01). (Neu + Eos) # accounted for 19.3% of total effect. To minimize the effect of LD and identify if TET2 and (Neu + Eos) # share a causal variant in a genomic region, we performed Bayesian Colocalization analysis. We found TET2 and (Neu + Eos) # colocalized with rs7705526, a telomerase reverse transcriptase (TERT) variant. (H4 = 0.997, Figure S2B and Table S5, Supporting Information).^[^ ^34^, ^35^ ^]^

### 3.5 TET2 Leads to Worse Ischemic Stroke Functional Outcome

**Figure 3:**
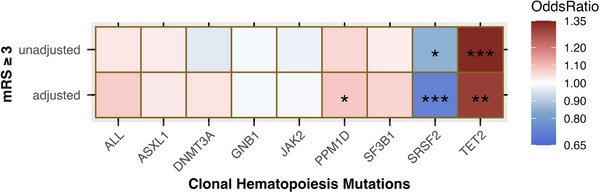
TET2 CH is associated with worse post‐stroke functional outcome. Gray box indicates insufficient SNPs for the analyses. The color gradient from blue to red indicates OR with blue being < 1, white being 1, and red being > 1. * indicates weak association (*p* ≤ 0.1) ** indicates moderate association (*p* ≤ 0.05), *** indicates strong association (*p* ≤ 0.01). N = 4363. P values are assessed by IVW test.

To assess if TET2 is associated with ischemic stroke recovery, we evaluated the effect of TET2 on 90‐day mRS. TET2 was strongly associated with mRS ≥ 3 (OR = 1.34, *P* = 0.005, **Figure** **3**; Table S10, Supporting Information). After adjusting for age, sex, initial stroke severity, and ancestry, TET2 remained moderately associated with adverse functional outcomes (OR = 1.30, *P* = 0.02. Interestingly, SRSF2 was protective against adverse functional outcomes (Unadjusted OR = 0.85, *P* = 0.05; adjustment OR = 0.72, *P* = 0.001; Figure 3; Table S10, Supporting Information). There were no heterogeneity and pleiotropy effects for the association between TET2 and adverse functional stroke outcome (Table S11, Supporting Information). Collectively, we showed that TET2 not only increased the risk of ischemic stroke but also worsened the functional outcome after a stroke, highlighting its clinical significance as a prominent emerging risk factor for stroke occurrence and recovery.

### 3.6 The Effect of CH on Hemorrhagic Stroke may be Mutation‐Specific

**Figure 4:**
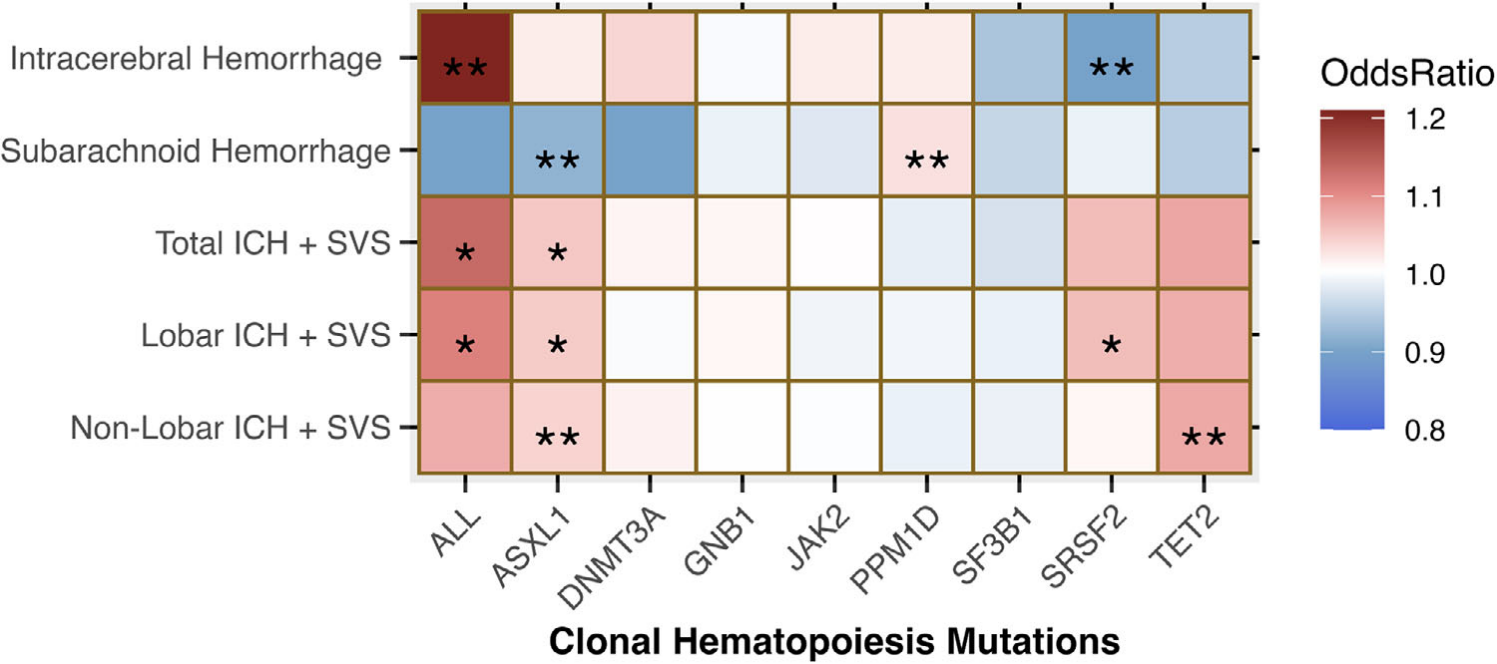
CH is implicated in hemorrhagic stroke. Gray box indicates insufficient SNPs for the analyses. The color gradient from blue to red indicates OR with blue being < 1, white being 1, and red being > 1. * indicates weak association (*p* ≤ 0.1) ** indicates moderate association (*p* ≤ 0.05), *** indicates strong association (*p* ≤ 0.01). N = 429209 for FinnGen Subarachnoid hemorrhage, N = 42909 for FinnGen intracranial hemorrhage, N = 239313 for Chung et al. P values are assessed by IVW test.

To interrogate the effect of CH driver mutations on hemorrhagic stroke and its subtypes, we used the FinnGen R10 consortium and Chung et al GWAS (**Figure** **4**; Tables S6 and S8, Supporting Information). Accounting for any CH mutation (ALL), there was a moderate association with intracerebral hemorrhage (ICH) (OR = 1.21, *P* = 0.02). In comparing the mutation‐specific associations, TET2 was moderately associated with non‐lobar ICH + SVS (OR = 1.07, *P* = 0.03) (Figure 4; Table S8, Supporting Information). PPM1D was moderately associated with nontraumatic subarachnoid hemorrhage (SAH) (OR = 1.02, *P* = 0.03). ASXL1 was moderately protective against SAH (OR = 0.92, *P* = 0.03) but moderately associated with greater non‐lobar ICH + SVS risk (OR = 1.04, *P* = 0.02). SRSF2 was moderately protective against ICH (OR = 0.90, *P* = 0.04). No heterogeneity or pleiotropy effects were identified for the association between CH and hemorrhagic stroke (Tables S7 and S9, Supporting Information). In sum, while CH is potentially implicated as a risk factor for hemorrhagic stroke, its mutation‐specific effects are not as consistent as those in ischemic stroke, warranting further studies.

## 4 Discussion

Recent studies have identified CH as a risk factor for cardiovascular and cerebrovascular diseases, independent of leukemic transformation.^[^ ^6^, ^7^, ^36^ ^]^ Our MR study, leveraging publicly available summary‐level large‐scale GWAS, provided robust statistical power and sample size to establish the relationship between CH, overall stroke, stroke subtypes, and stroke recovery. The scope of our study filled in the gap for associations previously unelucidated by observational studies. For example, we showed that SRSF2, a spliceosome mutation, is significantly associated with better ischemic stroke outcomes and lower risk of ICH. Additionally, we have independently replicated the main finding from two previous large‐scale cohort analyses on the association between TET2 and stroke.^[^ ^6^, ^7^ ^]^

TET2 loss‐of‐function was associated with higher rates of ischemic stroke. The strength of such association varied by stroke etiology.^[^ ^6^, ^7^ ^]^ We found that TET2 showed the strongest association with SVS, with a 29% increased risk, followed by LAS, with a 21% increased risk. However, no association was found between TET2 and CMS. These findings align with studies by Bhattacharya et al. and Arends et al., which reported an elevated risk of SVS and LAS in patients with any CH driver mutations. Additionally, TET2 loss‐of‐function mutation was associated with poor functional outcomes at 90 days, independent of age, sex, and initial stroke severity. Similarly, mutation in another epigenetic regulator, DNMT3A, was associated with functional disability, after adjusting for initial stroke severity, the use of intravenous thrombolysis or mechanical thrombectomy, and occurrence of hemorrhagic transformation.^[^ ^37^ ^]^

TET2 loss‐of‐function mutation may increase stroke risk and worsen outcomes through dysregulated hematopoietic cell function and chronic systemic inflammation. Our study found that nearly 20% of the effect of TET2 on SVS was mediated through neutrophils‐eosinophils count, suggesting that the aberrant activities of these cells as results of CH mutation acquisition may be partially responsible for TET2‐mediated SVS risk. Additionally, TET2 and neutrophils‐eosinophils count colocalized with rs7705526, a telomerase reverse transcriptase that regulates neutrophils.^[^ ^35^, ^38^ ^]^ Previous MR studies revealed that rs7705526 have longer genetically imputed leukocyte telomere length and are more likely to develop CH, such as a TET2 mutation. Thus, the colocalization result may suggest a higher chance of mutagenesis associated with longer telomeres rather than a direct effect of TET2 mutation.^[^ ^1^, ^39^ ^]^ Previous ex vivo studies have shown neutrophils with TET2 mutation displayed exacerbated inflammatory responses, further highlighting the role of pro‐inflammatory pathways in stroke etiology.^[^ ^38^ ^]^ TET2, as an epigenetic regulator, normally suppresses the transcription of inflammatory cytokines such as IL‐6 in macrophages.^[^ ^32^, ^40^ ^]^ This regulation prevented prolonged transcriptional activation and facilitated the resolution of inflammation. In animal studies, TET2 inhibition upregulated proinflammatory cytokine expression and exacerbated infarct volume.^[^ ^40^, ^41^ ^]^ Clinically, the presence of a genetic IL‐6 signal deficiency partially attenuated the risk of unfavorable functional outcomes and recurrent stroke in patients with CH^[^ ^7^, ^42^, ^43^ ^]^ Furthermore, functional TET2 may also exert its neuroprotective role by altering gene expression involved in cell junction, neuronal morphogenesis, and neurodevelopment pathways.^[^ ^41^ ^]^ Modulating TET2 function may present a promising avenue for suppressing neuroinflammation and associated tissue injuries.^[^ ^31^, ^32^, ^35^, ^40^, ^44^, ^45^, ^46^, ^47^ ^]^

Recognizing CH as a risk factor for ischemic stroke and TIA has several clinical implications. Hematologists should counsel patients with CH on their increased risk of both hematologic malignancy and ischemic stroke. It is crucial to assess and aggressively treat traditional vascular risk factors of stroke‐like hypertension, diabetes, and hyperlipidemia in CH patients to prevent compounded risk. Screening for CH or myeloproliferative malignancy in younger patients with unexplained ischemic stroke or TIA may be beneficial, with follow‐up by hematology.^[^ ^48^ ^]^ However, identifying individuals at high genetic risk for stroke must consider the potential psychological impact, equitable access to genetic screening, affordability, and safety of early intervention.^[^ ^49^, ^50^ ^]^ The association between CH and hemorrhagic stroke is less well‐defined. Although having any CH mutation is associated with 20% increased odds of ICH, no specific CH mutation elevated the risk of ICH alone. TET2 was associated with a higher combined risk of non‐lobar ICH and SVS, but not with lobar ICH and SVS, suggesting a shared pathomechanism of microvasculopathy behind non‐lobar ICH and SVS. Additionally, while previous research reported a higher prevalence of SAH among patients with any CH mutation,^[^ ^6^ ^]^ we found that the relationship between CH and SAH risk varied depending on the specific driver mutation. PPM1D was associated with elevated SAH risk, while ASXL1 lowered SAH risk. Such discrepancies between our work and others may partially explained by differences in databases used and methodology (Table S12, Supporting Information). There may also be allelic frequency and lifestyle differences between the cohort populations. The variability in the effect of each CH mutation on SAH suggests certain CH mutations may offer protective effects; however, given our modest effect size, these results should be interpreted with caution. Further research is needed to confirm these findings and understand any protective effects of CH on hemorrhagic stroke.^[^ ^6^ ^]^

Unexpectedly, we found SRSF2, a spliceosome mutation, was associated with favorable functional outcome at 90 days post stroke. Although the literature on SRSF2 and stroke is limited, few studies suggested that some CH mutations may offer protection against brain injury.^[^ ^51^, ^52^ ^]^ Investigating the specific pathomechanisms of individual CH mutations is crucial, as they may exert distinct effects on stroke outcomes.^[^ ^5^, ^6^, ^7^, ^53^, ^54^ ^]^

Despite the compelling evidence linking TET2 with stroke, several limitations remain in our study. First, we do not have access to individual‐level data to adjust for traditional stroke factors such as age, hypertension, hyperlipidemia, diabetes, and smoking status. Second, the phenotype of GWAS is already pre‐defined by the original study, limiting our ability to study specific subgroups. For example, we were unable to separate non‐lobar ICH from SVS to examine whether the effect of TET2 on non‐lobar ICH + SVS was driven by non‐ICH alone. The GWAS used for non‐lobar ICH + SVS has significant overlap with SVS GWAS from GIGASTROKE. Third, the precise mechanisms by which TET2 drives stroke risk need further elucidation, specifically how different mutations within the TET2 gene may confer varying levels of risk through animal models. A major future direction is to perform stroke models with primary TET2 deficient mice to validate the clinical and genetic findings in vivo. Lastly, our study is performed mainly with the European population so the results may not be generalizable to other ethnic and racial groups.

In conclusion, understanding the role of CH in stroke pathogenesis offers an opportunity to develop targeted interventions that could significantly reduce ischemic stroke incidence and improve outcomes for individuals with this genetic predisposition. Continued research and collaborative efforts will be essential in translating these findings into effective clinical strategies, ultimately enhancing stroke prevention and management in the era of genomic medicine.

## Conflict of Interest

The authors declare no conflict of interest.

## Author Contributions

S.L conceived the project, analyzed data, and co‐wrote the manuscript with input from all authors. Y.L provided intellectual input and genomic expertise. Y.W provided clinical expertise and funding, revised the manuscript, and supervised the entire project.

## Supporting information



Supporting Information

Supporting Information

## Data Availability

The data that support the findings of this study are available in the supplementary material of this article.
