# Supplementary material for: Multi‐Cohort Analysis Reveals Genetic Predispositions to Clonal Hematopoiesis as Mutation‐Specific Risk Factors for Stroke
Source: Adv Genet (Hoboken). 2025 Feb 8;6(1):2400047. doi: 10.1002/ggn2.202400047 (PMC11909397; doi:10.1002/ggn2.202400047)
Supplement: Supplementary file 1 — Supporting Information [file GGN2-6-2400047-s001.docx]

**Figure S1** Overview of the study design and data sources


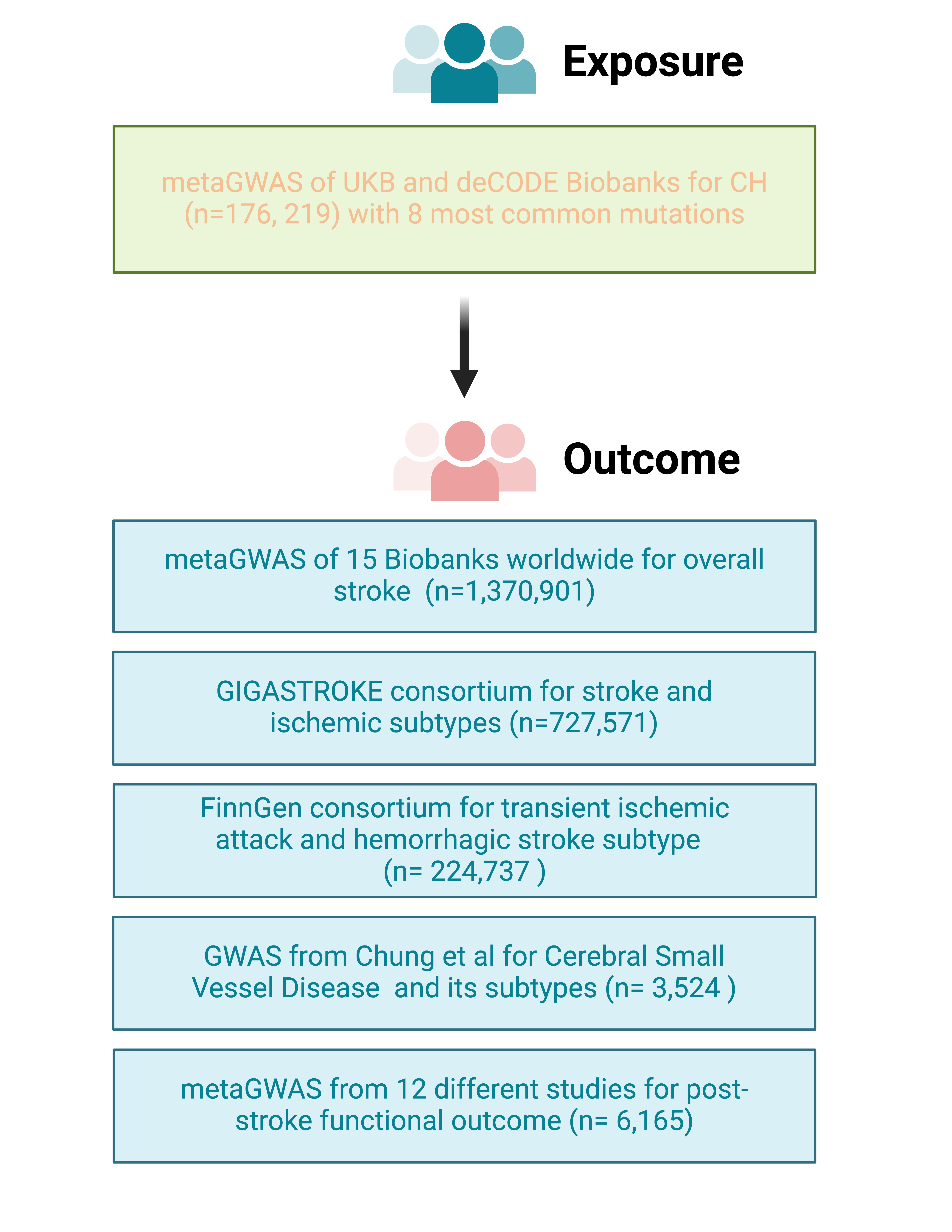


**Figure S2** Total Neutrophil and eosinophil counts partially mediate the effect of TET2 CH on small vessel stroke


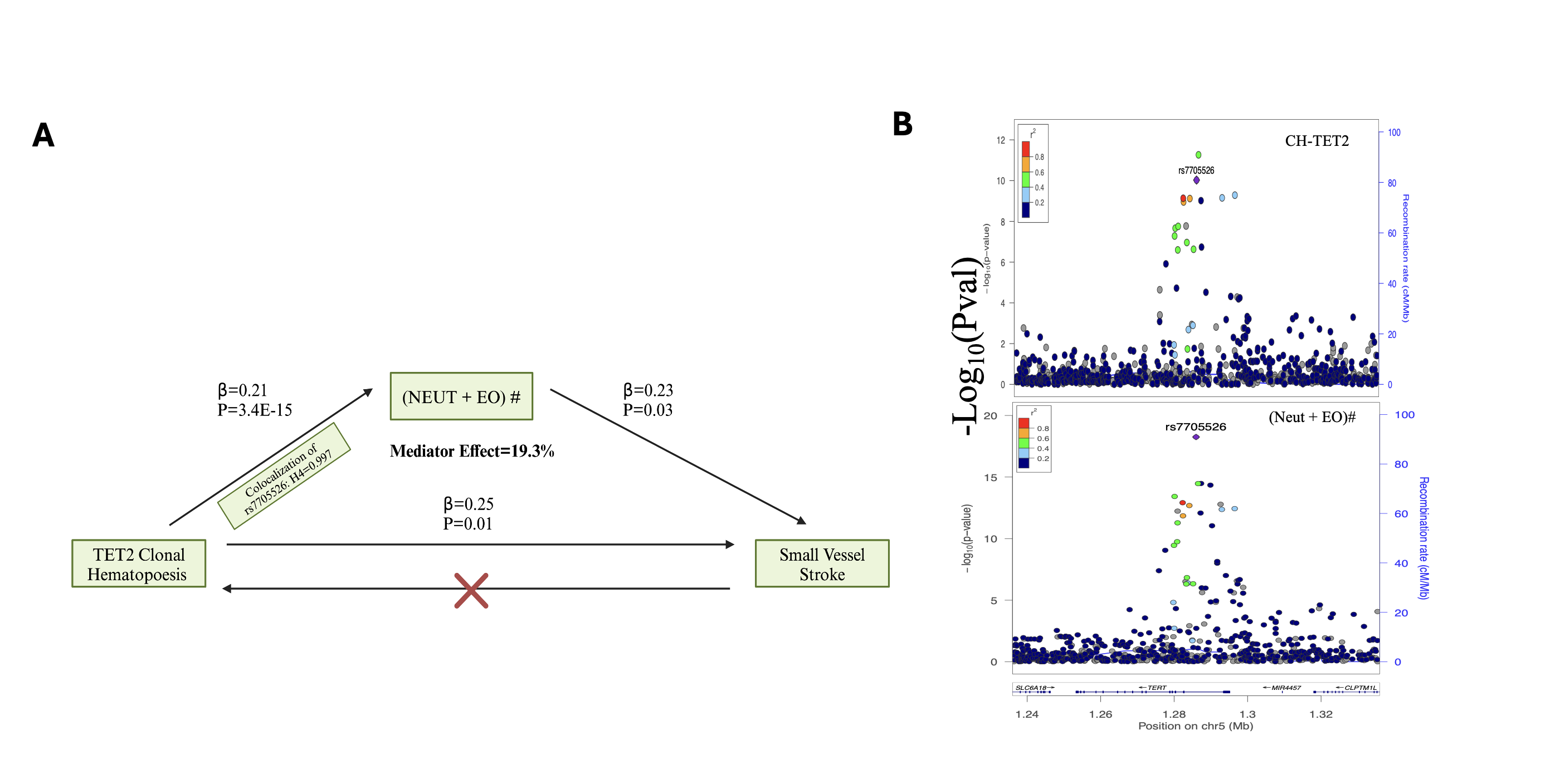


A: Forest plot and graphic scheme showing the result of two-step mediation analysis

B: Locus zoom plot showing the Colocalization results of TET2 CH and (Neu + Eos) #.
